# Histological and molecular characterization of bone integrity in osteogenesis imperfecta: a case series across genetic subtypes

**DOI:** 10.1093/jbmrpl/ziag111

**Published:** 2026-07-12

**Authors:** Zhiming Wu, Suzanne den Haan, Helen E King, Wouter H Nijhuis, Harrie Weinans, Anne J Spaans, Ralph Sakkers, Kelly Warmink

**Affiliations:** Department of Orthopaedics, University Medical Center Utrecht, Utrecht, 3584 CX, The Netherlands; Department of Orthopaedics, University Medical Center Utrecht, Utrecht, 3584 CX, The Netherlands; Department of Earth Sciences, Utrecht University, Utrecht, 3584 CB, The Netherlands; Faculty of Geosciences, University of Bremen, Bremen, 28359, Germany; Department of Orthopaedics, University Medical Center Utrecht, Utrecht, 3584 CX, The Netherlands; Department of Orthopaedics, University Medical Center Utrecht, Utrecht, 3584 CX, The Netherlands; Department of Orthopaedics, University Medical Center Utrecht, Utrecht, 3584 CX, The Netherlands; Department of Orthopaedics, University Medical Center Utrecht, Utrecht, 3584 CX, The Netherlands; Department of Orthopaedics, University Medical Center Utrecht, Utrecht, 3584 CX, The Netherlands

**Keywords:** osteogenesis imperfecta, collagen, bone mineralization, bone histomorphometry, raman spectroscopy

## Abstract

Osteogenesis imperfecta (OI) is a genetically heterogeneous skeletal disorder characterized by bone fragility and variable clinical severity. However, how molecular defects translate into alterations of bone microstructure and composition across OI subtypes remains incompletely understood. In this case series, we systematically evaluated cortical bone integrity in patients with OI types 1, 3, 4, 6, 8, and 14 using histological and molecular approaches, including Raman spectroscopy, and compared findings with non-OI controls. Histological analyses revealed case-specific disruption of bone architecture across OI cases, where the severity of bone disorganization increased progressively from OI type 1 to types 3, 6, 8, and 14. In addition osteocyte lacunar area (Ot.Lc.Ar) was increased specifically in OI subtypes 1, 6, 8, and 14 bones, while osteocyte lacunar appearance was heterogeneous in size, shape, alignment, and spatial distribution in OI types 3, 6, 8, and 14, underscoring the case-specific alterations. Consistently, polarized light microscopy demonstrated increased green birefringence under polarized light microscopy in OI types 1 and 14 and reduced lamellar thickness in OI types 1, 6, and 8. At the molecular level, Raman spectroscopic analyses showed reduced mineral and organic matrix signals in OI bone, specifically OI type 3, indicating compromised mineralization and altered bone matrix composition. Together, these findings illustrate the potential that OI bone phenotype illustrates potential subtype-specific trends in bone microarchitecture, collagen disorganization, impaired lamellar bone formation, and deficits in bone mineral and matrix composition. This integrative analysis links genetic defects in collagen-related and non-collagen genes to multiscale alterations in bone tissue, providing mechanistic insight into OI pathophysiology and highlighting potential structural targets for individualized therapeutic strategies.

## Graphical Abstract

**Graphical Abstract:**
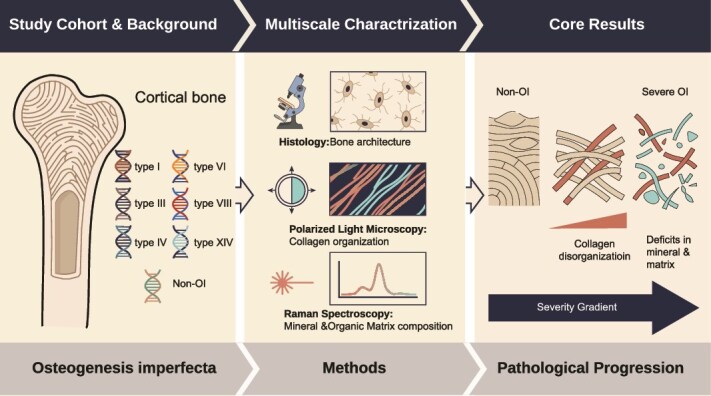

## Introduction

Osteogenesis imperfecta (OI), or brittle bone disease, represents a spectrum of genetic disorders characterized by an intrinsic bone fragility that leads to frequent fractures, bone deformities, and compromised quality of life.^1^ This condition is primarily caused by mutations in the genes responsible for the synthesis and structure of type I collagen,^2^ a protein critical to the development of bone strength and flexibility. Type I collagen forms a major component of the bone extracellular matrix, providing structural support and playing a central role in mineralization.^3^ Variations in the collagen structure due to these genetic mutations contribute directly to the unique bone phenotypes observed in OI patients. OI is divided into multiple clinical types, referred to by type 1 to type 23 according to the Online Mendelian Inheritance in Man. OI type 1, 3, and 4 are caused by mutations in the genes responsible for collagen type I synthesis, specifically COL1A1 and COL1A2.^4^ Type 1, the mildest form, generally involves frequent fractures but these patients have normal height and life expectancy, whereas more severe forms, such as type 8, result in greater skeletal deformities, growth deficiencies, and, in some cases, early mortality. OI type 4 is a moderate form of the several types of OI.^5^ Type 6, associated with mutations in the SERPINF1 gene, involves the production of pigment epithelium-derived factor by osteoblasts, which binds to collagen and serves as an anti-angiogenic factor.^6^ Clinically, type 6 OI presents with moderate to severe bone fragility and scoliosis. Histological examination reveals accumulation of osteoid within the bone tissue, along with a characteristic fish-scale pattern of bone lamellation.^7^ Type 8, associated with LEPRE1 mutations, disrupts the 3-hydroxylation of collagen type I. This form of OI can be severe to lethal, with patients often displaying significant growth deficiencies and skeletal deformities.^8^ Mutations in TMEM38B, encoding the TRIC-B channel responsible for calcium release from the endoplasmic reticulum, underlie type 14 OI.^9^ Clinically, type 14 presents with moderate to severe symptoms, with impaired hydroxylation of collagen, which is reduced in the helical region but increased in the telopeptides and increased retention of misfolded collagen within cells.^10^

Organized collagen fibrillar architecture confers anisotropic properties that are essential for tensile strength, elasticity, and directional load bearing. During bone formation, collagen fibrils mineralize with carbonated hydroxyapatite to form a composite with exceptional mechanical properties.^11^ This process involves nucleation and growth of minerals within the collagen matrix, regulated by factors such as citrate, which stabilizes amorphous calcium phosphate and promotes mineral deposition.^12^ Furthermore, cross-linking within collagen fibrils is a key determinant of bone quality. Enzymatic collagen cross-links, such as pyridinoline, enhance bone toughness, and resistance to fracture by improving matrix stability.^13^^,^^14^ Differences in collagen organization and the extent of bone remodeling can lead to variations in bone formation, potentially contributing to the phenotypic diversity observed among OI patients. Shapiro et al. demonstrated that more severe OI variants show greater persistence of woven bone and more immature structural patterns,^15^ likely due to structurally abnormal or insufficient collagen matrix that delays the maturation process. As the bone matrix matures, woven bone gradually transitions to lamellar bone once an adequate scaffold is established, a shift essential for achieving proper bone strength and structural integrity. In addition to disrupted bone matrix maturation processes, OI bone is also more highly cellular than normal bone.^16^ Increased cellularity in OI could be a compensation mechanism for defective collagen production, as the OI bone matrix often shows a higher turnover rate,^17^ with more osteoblasts (bone-forming cells) and osteoclasts (bone-resorbing cells). This imbalance in bone remodeling processes can lead to a net loss of bone mass and structural integrity, further exacerbating the fragility of the bones. Studies have shown that the increased porosity and disorganized collagen structure in OI bones are significant factors in reducing bone stiffness and strength, making them more susceptible to fractures.^16^ Understanding how collagen interacts with the bone microstructure and how these interactions differ between patients could shed light on the mechanisms underlying OI phenotypes. We hypothesized that patients with different forms of OI have arrangements that differ from non-OI bone both in their collagen alignment and mineral (Haversian bone) architecture.

To test this hypothesis, polarized light microscopy was used to evaluate collagen orientation, while Raman spectroscopy provided insights into the molecular composition of both the collagen and mineral phases. These complementary techniques enable correlation of structural and chemical parameters underlying bone fragility in different OI cases. By analyzing bone tissue from pediatric OI patients with different OI types and non-OI children undergoing surgery, we aimed to uncover differences in bone composition and microstructure of bone with variations in disease severities.

## Materials and methods

### Donors and tissue collection

Cortical bone samples were collected from human surgical waste in accordance with national and institutional ethical guidelines. Samples were obtained from 6 children with OI (mean age 8.33 yr, range 5-14 yr) and 2 non-OI controls (mean age 11 yr, range 3-19 yr) undergoing corrective osteotomy, immediately following surgical excision, all fresh cortical bone samples were placed in sterile tubes, and placed on dry ice in an insulated styrofoam box for immediate transport to the laboratory, and subsequently transferred to a −80 °C freezer for long-term storage without any chemical fixation. Before histological processing and Raman spectroscopic analysis, the samples were thawed at room temperature immediately before use. The anatomical origin of each cortical bone specimen was documented using the AO Pediatric Comprehensive Classification of Long Bone Fractures system, which hierarchically classifies fractures by bone (1 = humerus, 2 = radius/ulna, 3 = femur, 4 = tibia/fibula) and segment (proximal = 1, shaft = 2, distal = 3), with appended bone-specific lowercase letters indicating laterality (eg, l for left, r for right). Cortical bone sections were obtained from different anatomical regions of long bones, including proximal, shaft, and distal sites, as detailed in Table 1. Because histological processing is a destructive technique, these analyses were performed on a strategically selected subset derived from the broader Raman spectroscopy cohort. The selection criteria for histological measurements included: (1) physical size, preferentially selecting larger cortical bone fragments to ensure sufficient tissue volume for optimal decalcification and sectioning; (2) subtype representation, ensuring at least one representative sample from each available OI subtype; and (3) sample redundancy, prioritizing donors from whom multiple cortical bone fragments were available to preserve precious rare material. In accordance with ASBMR histomorphometric guidelines,^18^ parameters are reported using standardized nomenclature.

**Table 1:** Clinical characteristics and genetic information of study samples.

Sample	age	sex	OI Type	PCCF ^ 5^	Mutation	Reason for osteotomy	Bisphosphonate usage
**Non-OI 1**	19	F	\	33l, 43l	Achondroplasia	Corrective osteotomy of the left fibula and femur	\
**Non-OI 2**	3	M	\	43r	\	Corrective osteotomy of tibia due to abnormal tibial development	\
**OI Type 1**	9	M	1	32l, 41l	COL1A1: c.671G>A; p.(Gly224Asp)	Correction of bowing	Pamidronate
**OI Type 3**	5	M	3	31l, 33l, 42l, 32r, 42r	Mutation not known at parents’ request	Correction of severe bowing of the legs with a history of multiple fractures	Pamidronate and Zoledronate
**OI Type 4**	9	F	4	32r, 42r 42l 43l 31 re	COL1A1: c.617G>A; p.Gly206Asp	Correction of bowing	Pamidronate and Zolendronate
**OI Type 6**	7	M	6	\	SERPINF1: c.205C>T; p.(Arg69) and c.886G>A; p.(Glu296Lys)	Correction of severe bowing of the legs and fracture fixation	Pamidronate
**OI Type 8**	14	F	8	\	P3H1: c.2055+86A>G; p.(?) (homozygous) and C1orf210: c.1A>G; p.(Met1?) (homozygous)	Placing nail right femur	Zolendronate
**OI Type 14**	6	F	14	\	Homozygous pathogenic variant of THEM38B gene and mosaic Turner combination 46xx with 45× cell lines 46	Correction of severe bowing of the legs	Risedronate

Summary of age, sex, OI subtype, anatomical origin of cortical bone samples (PCCF), reason for osteotomy, bisphosphonate usage, and identified genetic variants for all individuals included in the study. The cohort included pediatric patients with OI types 1, 3, 4, 6, 8, and 14, as well as non-OI controls. The samples listed in this table represent the primary subset strategically selected to undergo both histological and Raman spectroscopic analyses. Pathogenic or putative disease-associated variants were detected in collagen-related genes (*COL1A1*) and non-collagen genes associated with recessive or rare OI forms, including *SERPINF1, P3H1* (*LEPRE1*), and *TMEM38B*. One OI type 3 case had an undetermined genetic mutation at the request of the parents. One non-OI sample was diagnosed with achondroplasia.

Abbreviations: F, female; M, male.

### Masson’s trichrome staining

After fixation, cortical bone specimens were decalcified in OSTEOMOLL decalcifying solution (Merck Millipore) at room temperature with gentle agitation. The solution was refreshed regularly according to the manufacturer’s instructions until complete decalcification was achieved, which was verified by needle testing. Following decalcification, samples were thoroughly rinsed prior to routine dehydration and paraffin embedding. Tissue sections of 5 μm thickness were deparaffinized using Histoclear/xylene (6 min initially, followed by 1-min washes) and rehydrated through graded ethanol (3-min initial step, 5-s washes, and a final 2-min step) before rinsing in distilled water. Slides were incubated in Bouin’s fluid at 56 °C for 1 h, rinsed in tap water until the yellow color disappeared, and stained sequentially with Weigert’s hematoxylin (10 min), Biebrich Scarlet Acid Fuchsin (10 min), phosphomolybdic-phosphotungstic acid (15 min), aniline blue (10 min), and 1% acetic acid (1 min). Slides were washed thoroughly with distilled water between steps, dehydrated with graded ethanol and Histoclear/xylene, and mounted with Eukitt mounting medium.

### Polarized light microscopy observation and Picrosirius red staining

To evaluate collagen organization and orientation, bone sections were stained with 0.1% Picrosirius red solution for 1 h to achieve near-equilibrium staining. Picrosirius red dyes are elongated birefringent molecules, when bound within the upper groove of well oriented collagen molecules that are eosinophilic, the dyes orient parallel to the long axis of each collagen fiber, thereby greatly enhancing the normal birefringence stemming from a uniform fiber distribution.^19^ Birefringence is a measure of the difference in the refractive indices of 2 separate components (the fast ray and slow ray) of the incident ray passing through a sample.^20^ In the phenomenon of birefringence, as light enters anisotropic compounds or structures, the light beam is split into 2 beams with different velocities whose vibration planes are perpendicular to each other.^21^ Polarized light microscopy enables the visualization of anisotropic structures by exploiting differences in light propagation through materials with direction-dependent properties. This technique exploits the stoichiometric binding of Sirius red to the collagen triple helix, allowing distinction between mature, organized collagen and immature, disorganized tissue. Under polarized light, mature collagen with aligned fibers shows strong red-yellow birefringence (anisotropy), whereas immature collagen with randomly oriented fibers displays weak greenish birefringence. Brilliant red-yellow fibers result from the interaction of the molecules with type I collagen; in contrast, type III collagen is weakly birefringent and characterized by thin greenish fibers.^22^ Tissue sections were deparaffinized, rehydrated, and nuclei were counterstained as described above. Following 1 h in Picrosirius red solution, slides were rinsed sequentially in 0.1% acidified water for 1 min and 0.5% acidified water for 1 min. Dehydration and mounting were performed as described above. Collagen fiber alignment was assessed using polarized light microscopy (Olympus BX43 equipped with a 360° Polarizer U-AN360P) with the polarizer set at 0° and 45°, to evaluate collagen birefringence. Merged images were generated for analysis.

### Histology image quantification

Image quantification was performed using Fiji software (NIH) to analyze collagen birefringence and nuclear area. Threshold adjustment was applied via “Image > Adjust > Threshold” to selectively isolate collagenous structures, with thresholded regions visually confirmed by red highlighting based on the region of interest (blue/red staining area for Masson’s trichrome staining; cell nuclear area for H&E staining; yellow/red/green birefringence area for Picrosirius red-staining). Measurement parameters were configured using “Analyze > Set Measurements,” ensuring both “Area” and “Limit to Threshold” options were enabled. The Limit to Threshold setting restricted quantification exclusively to the thresholded regions, preventing inclusion of region of noninterest. Final measurements were obtained using “Analyze > Measure,” yielding birefringent area values in percentage. Triplicate measurements per sample were averaged to ensure reproducibility.

### Raman spectroscopy measurements

Undecalcified cortical bone samples were analyzed without fixation, embedding, or decalcification. The clinical characteristics and anatomical origins of these samples are detailed in Table 2. Specimens were thawed from −20 °C and placed directly on glass slides, orientated perpendicular to the objective lens for Raman measurements to preserve the native mineral–matrix composition and minimize preparation-induced artifacts. However, as the cortical bone samples consisted of irregular fragments, the long axis could not be reliably defined, and their orientation relative to the slide therefore remains uncertain. In total, 38 cortical bone samples from non-OI controls and 15 from OI patients were analyzed. All OI donors had received bisphosphonate treatment. Raman spectroscopic analysis of the bone samples was performed using a WITec alpha300 R confocal Raman microscope (WITec). The excitation source was a 532 nm laser, operating at a power of 40 mW. A 50× long working distance objective lens (Zeiss) with a numerical aperture of 0.55 was used to measure the samples. Spectroscopic imaging was conducted with a lateral and depth resolution of ~1 μm. To account for the intrinsic heterogeneity of bone tissue, Raman data were acquired using a grid-based mapping approach rather than single-point measurements. Newly deposited osteoid regions were avoided according to their Raman fluorescence.^23^ Maps were collected with a step size of 10 μm, covering predefined regions of interest within the cortical bone matrix. Regions selected for analysis were chosen to represent the surrounding bone matrix while avoiding large vascular canals, cracks, and preparation artifacts. Within each mapped region, spectra were acquired across both relatively homogeneous and heterogeneous areas of the tissue to capture local variability in matrix composition. Representative mapping regions are shown in Figure S1. This mapping-based acquisition strategy has been recommended in previous studies to reduce selection bias and improve the reproducibility of Raman measurements in bone tissue, where local composition can vary substantially depending on tissue maturity and microstructural organization.^24^ A spectral grating of 600 grooves/mm was used to limit the time required to obtain a spectrum and thus exposure of the sample to the laser. The exposure time for each pixel was set to 1 s. For data acquisition, the spectral data were truncated between 300 and 1800 cm^−1^ and subjected to background correction using the shape function (filter size 300) available in the Project 5 software (WITec). This software was also used for band extraction and further analysis.

**Table 2:** Clinical characteristics and anatomical origin of additional bone samples utilized exclusively for Raman spectroscopy.

Sample	Age	Sex	OI Type	PCCF ^ 5^	Mutation	Reason for osteotomy	Bisphosphonate usage
**1**	14	F	III	43	COL1A1: c.1113G>A; p.Gly154Arg	Unknown	Pamidronate
**2**	19	M	III	13	Unknown	Unknown	Unknown
**3**	5	F	IV	32l, 32r	COL1A2: p.Gly103del	Alignment of both femora	Pamidronate
**4**	20	M	IV	42l	COL1A2: c.1009G>A; p.Gly337Ser	Osteotomy fully according to current guidelines	No
**5**	5	F	III	43r	COL1A2: c.2967_2984dup	Unknown	Actonel
**6**	3	F	III	42l, 42r	COL1A1: c.1715G>C; p.Gly572Ala	Tibia two small pieces	Pamidronate
**7**	3	M	VIII	12l	LEPRE1: c.628C>T; p.Arg210Ter	Correction osteotomy tibia	Zoldronate acid
**8**	11	F	IV	41, 43l	COL1A1: c.617G>A; p.Gly206Asp	Correction osteotomy tibia	Zoldronate acid
**9**	8	F	III	NA	NA	OI transplant	Yes
**10**	14	M	I	NA	COL1A1: c.671G>A; p.(Gly224Asp)	Placing 8-plate	NA
**11**	14	M	NA	32	NA	Segment resection femur	NA
**12**	12	F	NA	11	NA	unknown	NA
**13**	7	M	NA	41l	NA	Stüve-Wiedemann (syndrome)	NA
**14**	2	F	NA	43r	NA	Fibula aplasia type C for which Syme (amputation)	NA
**15**	5	F	NA	43r 42li	NA	Fibula aplasia	NA
**16**	19	M	NA	31l,31r	NA	Femur osteotomy surgery (OR) was scheduled/recorded on 01-01-10, but was actually 23-06-2005	NA
**17**	13	M	NA	31l	NA	DVO (derotation varization osteotomy) for perthes	NA
**18**	12	M	NA	N/A (talus, bilateral)	NA	Talectomy left and rightl, Talus due to arthrogryposis	NA
**19**	15	M	NA	N/A (Chopart joint, right)	NA	Triple arthrodesis due to clubfeet	NA
**20**	13	M	NA	42l	NA	Fibula aplasia	NA
**21**	11	F	NA	31l	NA	Sharrard (procedure), tendon transfer feet, Pemberton (osteotomy)	NA
**22**	12	M	NA	43r	NA	Lengthening fibula resection	NA
**23**	16	M	NA	33l	NA	Distal femoral extending osteotomy	NA
**24**	Unknown	Unknown	NA		NA	Unknown bone, possibly femur	NA
**25**	16	M	NA	31 left	NA	Subtrochanteric osteotomy due to perthes	NA
**26**	13	M	NA	N/A (Tarsal, right)	NA	Atypical clubfeet	NA
**27**	14	F	NA	33l	NA	Femur osteotomy distal left	NA
**28**	3	M	NA	43r	NA	Anlage (rudiment) and tibia	NA
**29**	7	M	NA	42l	NA	Fibula in stump tibia specimen: fibula also osteotomy tibia end	NA
**30**	2	F	NA	31l	NA	Open reduction, DVO, Pemberton left	NA
**31**	27	F	NA	N/A (foot)	NA	Boyd (amputation) right	NA
**32**	13	F	NA	31l	NA	Salter (osteotomy) left and DVO left	NA
**33**	1	F	NA	N/A (metatarsal/phalanges)	NA	Polydactyly toe left	NA
**34**	8	F	NA	43r	NA	Dome (osteotomy) right	NA
**35**	4	F	NA	31r	NA	DVO proximal femur right	NA
**36**	19	F	NA	43r, 33r	NA	Achondroplasia Dome right AO 43 fibula and distal femur AO 33 osteotomy right	NA
**37**	18	F	NA	21r	NA	Ulna and radius osteotomy	NA
**38**	15	M	NA	N/A (MT5, bilateral)	NA	MT (metatarsal) 5 base osteotomy	NA
**39**	18	M	NA	NA	NA	Precise nail right tibia and osteotomy right fibula	NA
**40**	14	M	NA	NA	NA	Epiphysiodesis knee	NA

This table details the additional cortical bone fragments derived from the donor cohort that were subjected only to Raman spectroscopic mapping (without histological processing). The summarized data includes age, sex, OI subtype, anatomical origin of the specific bone fragments (PCCF), reason for osteotomy, and bisphosphonate usage. Utilizing these additional fragments allowed for a broader assessment of compositional variance within the cohort while preserving tissue for destructive histological analyses.

Abbreviations: F, female; M, male; PCCF, Pediatric Comprehensive Classification of Long Bone Fractures.

### Band selection and quantification

**Figure 1:**
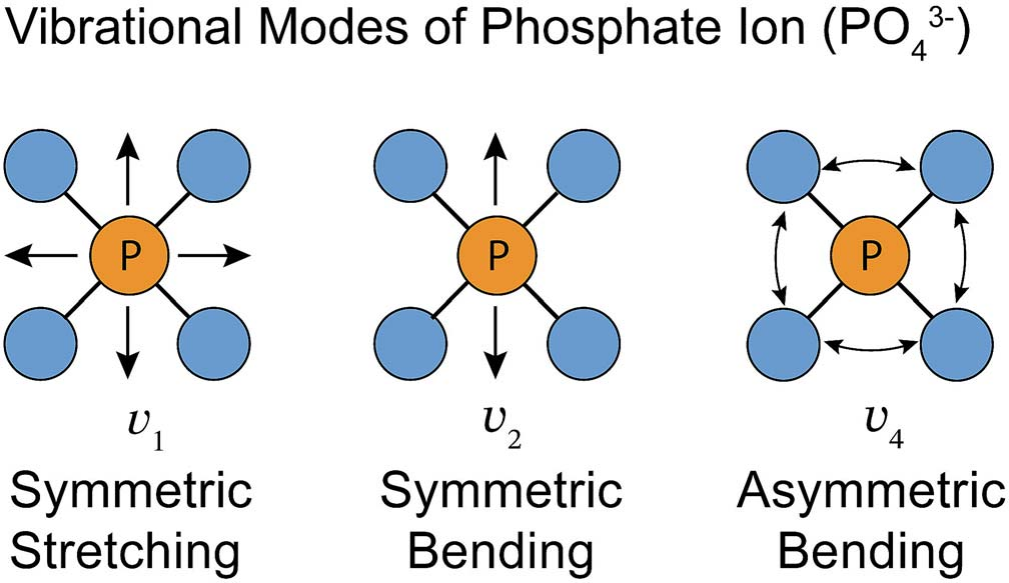
Vibrational modes of the phosphate ion (PO₄^3−^). Schematic representation of the major vibrational modes of the phosphate tetrahedron detected in Raman spectroscopy: including the ν₁ symmetric stretching, ν₂ symmetric bending, and ν₄ asymmetric bending modes. These characteristic vibrational patterns underlie the Raman spectral peaks commonly used to assess phosphate structure and mineral phases. The ν₁ mode is particularly sensitive to the transition from amorphous calcium phosphate to crystalline hydroxyapatite, while the ν₂ and ν₄ bending modes contribute to identifying phosphate environments associated with mineralization and extracellular matrix maturation.

The collagen quality parameter used in Raman spectroscopy is derived from a subset of partially resolved amide I bands (peptide bond C==O stretch, ~1666 cm^−1^),^25^ which is especially sensitive to secondary structures.^26^ The two bands at 1245 and 1268 cm^−1^ have been variously assigned to distinct amide III^27^ vibrations, which involves N-H bending and C-N stretching vibrations and provides complementary information of 2 different secondary structures (random coil and alpha or triple helix Matrix bands).^28^ In addition to the amide bands, the ~CH_2_ bending vibrations, often observed around 1450 cm^−1^, are indicative of the presence and motion of methylene groups within the collagen structure. These vibrations can reflect changes in the molecular environment and packing of collagen fibers, which are crucial for understanding the mechanical properties and functional state of the tissue. For instance, modifications such as glycation or cross-linking can lead to shifts in these Raman bands, providing insights into the biochemical changes occurring within the collagen matrix (~CH_2_ at 1450 cm^−1^).^24^^,^^29–32^ In Raman spectroscopy, the phosphate ion (PO₄^3−^) exhibits characteristic vibrational modes that provide critical insights into its structural environment. Among these, the ν₁, ν₂, and ν₄ bands are commonly analyzed. As shown in the Figure 1, the ν₁ mode (960 cm^−1^) corresponds to the symmetric stretching vibration, where all 4 P–O bonds expand and contract simultaneously in a coordinated and symmetrical manner. This results in a breathing-like motion of the PO₄ tetrahedron. The ν₂ mode (431 cm^−1^) represents symmetric bending, involving simultaneous changes in the internal O–P–O bond angles while maintaining symmetry, and this is a coordinated angular deformation of the tetrahedral structure. The ν₄ mode (590 cm^−1^), known as asymmetric bending, also involves bond angle deformation, but in a less uniform fashion, with some angles increasing while others decrease. These vibrational movements describe how atoms within the phosphate group dynamically shift relative to each other, and they are highly sensitive to local factors, such as crystal structure, cation substitution, and hydration. The ν1 mode, corresponding to the symmetric stretching of the phosphate group, is a critical indicator of the presence of bioapatite, as demonstrated in studies involving bone analysis using Raman spectroscopy.^33^ This mode is also observed in the transformation of amorphous calcium phosphate to crystalline hydroxyapatite, where a peak shift to 960 cm^−1^ signifies the formation of the crystalline phase.^34^ The ν2 and ν4 modes, associated with bending vibrations, are equally significant in characterizing phosphate minerals. For instance, the ν2 mode at 431 cm^−1^ and the ν4 mode at 590 cm^−1^ have been identified in the Raman spectra of dental pulp stem cells during osteogenic differentiation, highlighting their role in monitoring extracellular matrix changes.^35^ Bands of interest included: phosphate ν1 (960 cm^−1^), assessed over a 40 cm^−1^ region, was used to evaluate mineralization levels. Phosphate ν2 (431 cm^−1^) and phosphate ν4 (590 cm^−1^). The intensity contribution from these bands was estimated using a filter with a spectral width of 80 cm^−1^. Amide I (1666 cm^−1^) and Amide III (1250 cm^−1^), with filter widths of 120 and 80 cm^−1^, respectively, were used to assess collagen composition. In addition, the CH₂/CH_3_ band (typically around 1450 cm^−136^also known as δ(–CH₂) or δ(–CH₃)), analyzed over an 80 cm^−1^ region, in biological tissues (including bone, skin, and cells), this band reflects the presence and environment of alkyl chains (methylene, methyl groups) –CH₂/–CH₃^36^ in organic components (such as proteins, collagen, and lipids). Since the –CH₂/–CH₃ band is mainly located in lipid chains or protein side chains, the intensity and morphology of this band can reflect: the content or concentration of the organic matrix (eg, the amount of collagen or lipids); the conformation or environment of the chain segments (eg, the density of alkyl chains, aggregation state, and interaction with water or minerals); in Raman/IR studies of bone tissue, it can serve as an indicator of “organic matrix composition” or “alkyl chain status.”^37^ If the collagen fibers in the bone tissue are well-organized and the organic matrix content is high, the –CH₂/–CH₃ group may be in a highly organized and dense state, and its corresponding 1450 cm^−1^ band may appear stronger, more symmetrical, or narrower. Conversely, if the collagen fibers are sparse and disordered, then the alkyl chains may be more “loose,” and their vibrational environment may be more heterogeneous, potentially leading to a decrease in intensity, broadening, or slight shift in position of the 1450 cm^−1^ band. To account for variations in mineralization across samples, the band center of Phosphate ν1 was determined by using the center of mass within the specified region. Histograms were generated by plotting values from 957 to 963 cm^−1^ in 100 bins of equal width. A pure collagen spectrum was subtracted to minimize the impact of non-collagenous components, and normalization was performed with reference to the Amide III (1250 cm^−1^) band. Non-collagenous contributions were further filtered using a mask based on the proline vibration at 858 cm^−1^, excluding pixels representing pure water or poly-aspartic acid, while preserving those of mineralized and unmineralized collagen.

### Statistical analysis

Data are presented as mean ± SD, unless otherwise stated. Normality of data distribution was assessed using the Shapiro–Wilk test. For comparisons between two groups, an unpaired 2-tailed Student’s *t-*test was used for normally distributed data, while the Mann–Whitney U test was applied for non-normally distributed data. For comparisons involving more than 2 groups, one-way analysis of variance (ANOVA) followed by Tukey’s post hoc test was used when data met assumptions of normality and homogeneity of variance; otherwise, the Kruskal–Wallis test with Dunn’s multiple comparisons correction was applied. All analyses were performed using GraphPad Prism (version 10.6.1; GraphPad Software). For Raman measurement analyses, OI samples were pooled into a single group because the number of specimens within individual OI subtypes/genotypes was insufficient for statistically robust subgroup analyses. This approach was used to explore overall disease-associated differences relative to controls, while acknowledging the biological heterogeneity of OI.

### Study design

This study was designed as a human exploratory case series using cross-sectional analysis of cortical bone tissue obtained from pediatric patients with OI and non-OI controls. The primary objective was to characterize alterations in bone microstructure and molecular composition across genetically distinct OI cases using histological, polarized light, and Raman spectroscopic analyses.

#### Primary outcome

The primary outcome was the difference in cortical bone collagen organization and mineral–matrix composition between OI cases and non-OI controls, assessed through: collagen birefringence (polarized light microscopy after Picrosirius red staining). Bone matrix mineral and organic signatures (Raman spectroscopy: ν1 phosphate peak position, amide I/III, CH₂~/CH₃~ band).

#### Secondary outcomes

Histological evaluation of lamellar vs woven bone distribution (Masson’s trichrome), quantification of osteocyte lacunar number per bone area, comparative structural abnormalities across genetic OI cases (types 1, 3, 4, 6, 8, 14).

#### Study population and sample size considerations

Cortical bone samples were obtained from 6 children with confirmed OI and 2 non-OI controls undergoing corrective osteotomy. Due to the extreme rarity of several OI subtypes included (eg, types 6, 8, and 14), a formal power calculation was not applicable. Therefore, this study is presented as an exploratory case series aimed at illustrating potential structural and compositional changes in bone across different OI types, rather than drawing definitive statistical conclusions between subtypes.

#### Assumptions underlying sample size

Effect sizes were expected to be large due to known pathological differences between normal bone and severe OI subtypes. Raman and histology outcomes are continuous with high spatial replication (hundreds–thousands of spectra per sample), increasing the effective analytical power despite limited donor numbers. The design aimed to establish patterns and mechanistic signatures, not prevalence estimates.

#### Study overview

All samples were processed using harmonized histological protocols, and analyses were performed using standardized image quantification and Raman spectral extraction workflows. Investigators conducting image quantification and Raman analysis were blinded to OI subtype to reduce interpretation bias. This integrated structural–molecular design enabled multiscale assessment of bone integrity across a spectrum of OI severities.

### Type of study

Because this work involves human surgical tissue and observational comparisons without interventions, it qualifies as: a human observational cross-sectional study.

## Results

### Variations in cortical bone architecture among OI cases observed by Masson’s trichrome staining

**Figure 2:**
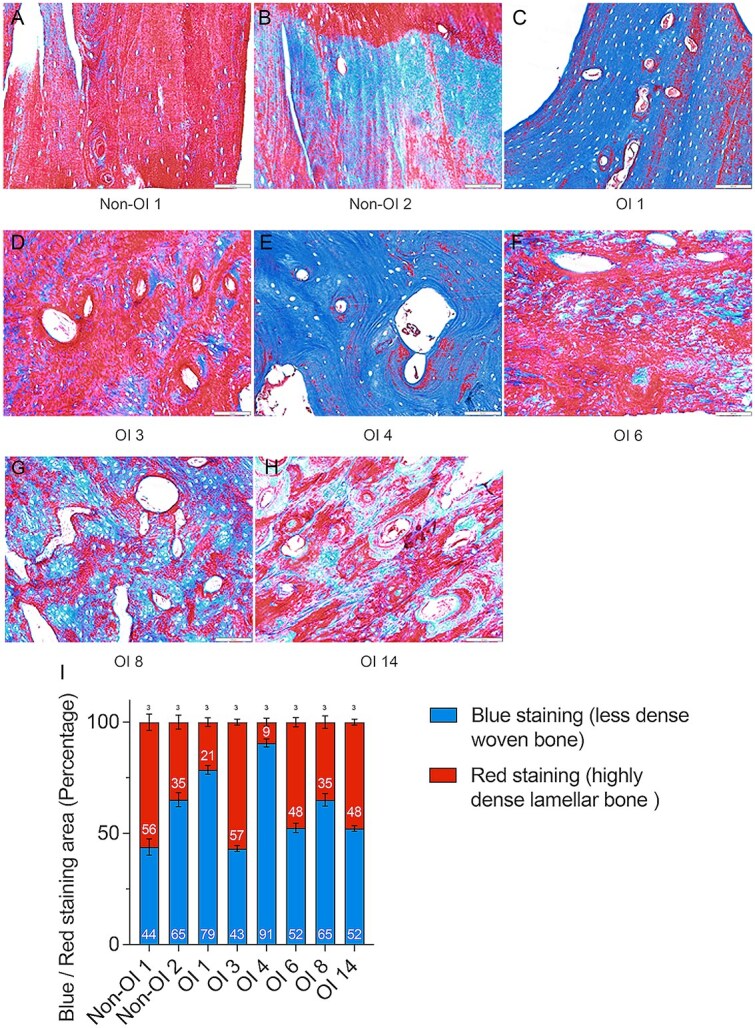
(A-H) Representative Masson’s trichrome-stained bone sections from non-OI and OI individuals (scale bars are 100 μm). Lamellar bone is stained red, while osteoid tissue or less densely packed mineralized woven bone tissue appears blue. Non-OI bone (Figure 2A and B) shows relatively more continuous and organized lamellar regions compared to OI samples, although substantial areas of woven bone are also present, whereas OI samples exhibit increased woven bone (particularly in type 1 and type 4) and a highly disorganized pattern with red and blue fragments in type 3, 6, 8, and 14. (I) Quantifying blue and red staining area (in percentage) for non-OI and OI samples. Data are presented as mean ± SD of 3 technical replicates.

Masson’s trichrome staining revealed marked visual differences in tissue architecture (Figure 2). A blue reaction was localized to osteoid tissue and woven bone, whereas a red reaction indicated more tightly packed lamellar bone.^38^ One non-OI samples demonstrated well-organized and densely packed lamellar bone (Figure 2A). In the non-OI 2 sample, red- and blue-stained areas showed a clear separation, suggesting that differences in tissue density affected dye retention in different regions (Figure 2B). This finding was likely related to age-dependent bone remodeling, as this sample was obtained from a 3-yr-old donor. Tissues from OI type 1 and type 4 showed minimal and irregularly distributed red staining, indicating a marked reduction in lamellar bone, together with abundant blue-stained areas representing less densely packed woven bone (Figure 2C, E, and  I). The OI type 1 bone sample also contained an increased number of osteocyte lacunae (Figure 2C). In OI type 3 (Figure 2D), collagen fibers appeared disorganized, with patches of lamellar bone (red) interspersed with regions of lower density woven bone (blue). OI types 6, 8, and 14 (Figure 2F-H) showed pronounced architectural disorganization, characterized by fragmented, red-stained lamellar bone interrupted by blue-stained woven bone. In OI type 6 (Figure 2F), a characteristic fish-scale lamellar pattern was observed, accompanied by severe structural deterioration. OI type 8 samples exhibited large fragments of blue-stained woven bone with a high concentration of osteocyte lacunae (Figure 2G). OI type 14 samples showed similarly profound abnormalities in lamellar and woven bone organization, with extensive low-density woven bone regions (Figure 2H). Increased intracortical porosity was observed across all OI cortical bone samples.

### Increased osteocyte lacunar number per bone area in OI assessed by H&E staining

**Figure 3:**
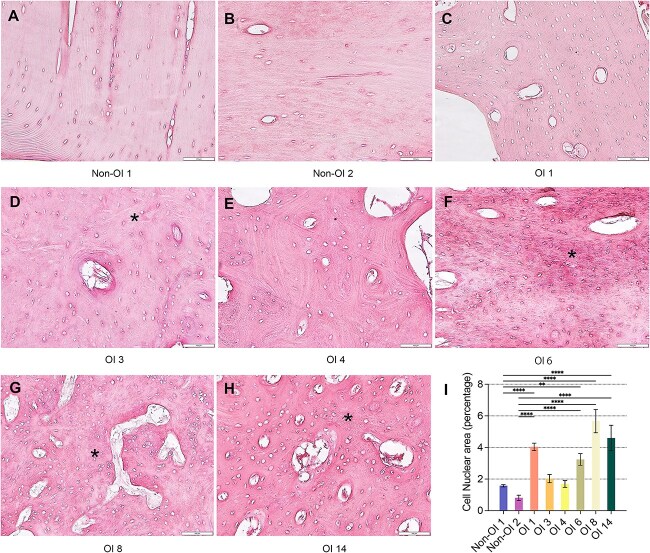
Histological assessment of bone structure and osteocyte organization in non-OI and OI samples. (A-H) Representative H&E stained sections of non-OI and OI bone samples. Asterisks (^*^) indicate regions showing irregular, non-aligned and discontinuous matrix patterns. Scale bars: 100 μm. (I) Quantification of osteocyte lacunar area (in percentage) for non-OI and OI samples. Data are presented as mean ± SD of technical replicates.

H&E staining further highlighted structural differences. Osteocyte lacunar area was used as a two-dimensional descriptor of osteocyte cellularity rather than a stereological measure of osteocyte lacunar number per bone area. In non-OI bone samples (Figure 3A and B), osteocyte lacunar appear regularly shaped, uniformly distributed, and aligned within the bone matrix. OI type 1 samples (Figure 3C) retained visible canaliculi and relatively well-packed lamellar regions. In contrast, in OI types 3, 6, 8, and 14 (Figure 3D and F-I), osteocyte lacunar show increased variability in size, shape, and spatial distribution, with occasional clustering and heterogeneous matrix appearance. OI type 1, 6, 8, and 14 bones show a significant increase in lacunar area compared to non-OI controls, indicating higher osteocyte lacunar number per bone area in OI bone (Figure 3I).

### Collagen organization and lamellar thickness in OI cortical bone detected by polarized light microscopy

**Figure 4:**
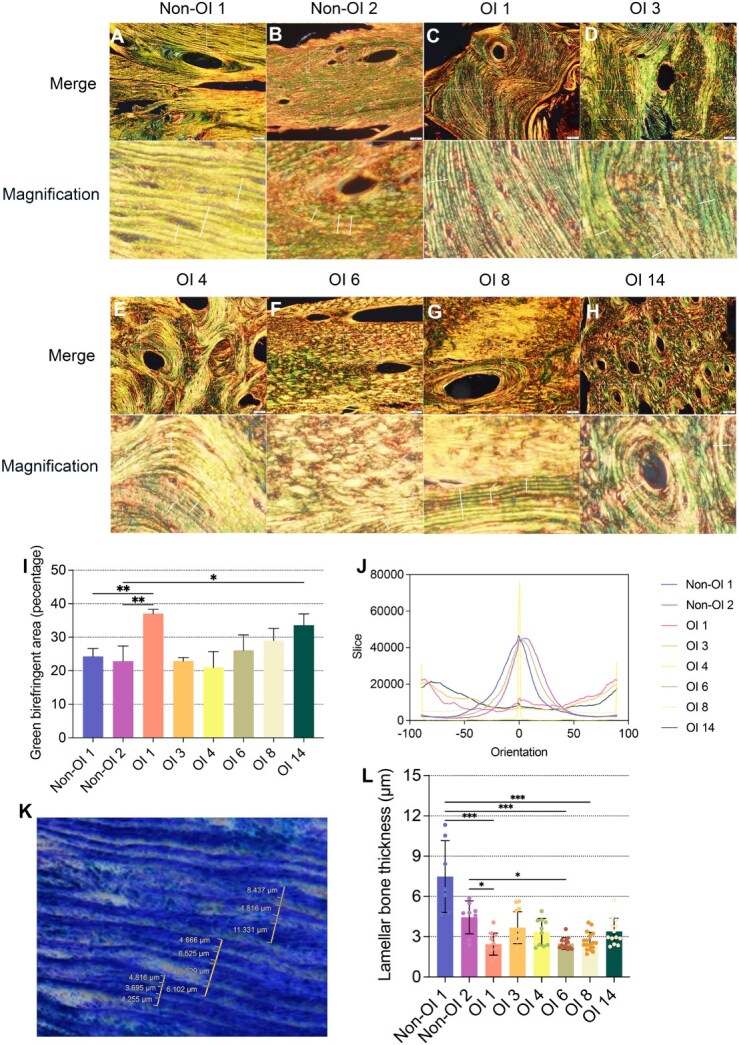
Collagen organization in non-OI and OI cortical bone using polarized light microscopy with Picrosirius red staining. Birefringence colors indicate collagen fiber organization: yellow-red represents thick, mature collagen fibers, while green represents thinner, less organized collagen fibers. (A-H) Representative merged polarized light microscopy images of Picrosirius red-stained bone sections from non-OI and OI individuals. The individual polarization angle images (0° and 45°) and their corresponding collagen fibril orientation analyses (HSV pseudo-colored images) are provided in Figure S2. The dotted frame in the merged image is enlarged and placed in the “Magnification” row. White lines indicate the lamellar bone regions selected for lamellar thickness measurements and define the corresponding regions of interest. Scale bars: 50 μm. (I) Percentage of green birefringent area in non-OI and OI samples. (J) Polar plot showing collagen fiber orientation distribution from picrosirius red-staining image processed by OrientationJ of the direction row. OrientationJ’s HSV encoding (hue: fiber angle, saturation: coherency) preserves original stain colors. Different curves represent angular slice counts across sample groups, highlighting variations in fiber alignment. (K) Illustration of a non-OI bone sample zoomed in with color inversion to illustrate the lamellar bone being measured. (L) Quantification of the thickness of lamellar bones of white lines. (^****^p < .0001, ^**^p < .01, ^**^p < .05, compared to non-OI). Data are presented as mean ± SD of technical replicates.

Collagen organization showed case-dependent alterations in OI cortical bone (Figure 4A-H). While differences in birefringence patterns were observed qualitatively, no significant differences in the percentage of green birefringent area were detected between non-OI samples and several OI cases, apart from OI type 1 which was significantly increased compared to non-OI controls, and OI type 14, which was increased compared to non-OI 2 (Figure 4I). Lamellar bone thickness was significantly reduced in OI types 1, 6, and 8 compared to non-OI 1, and in OI types 1 and 6 compared to non-OI 2 (Figure 4K and L). Notably, substantial variability was also observed between the 2 non-OI samples (Figure 4L). OI type 1 samples exhibited substantial thinning and decreased packing density of collagen fibers, accompanied by increased green birefringence (Figure 4C, I, J, and  L). The collagen fibers also appeared wavier, consistent with the minimal red staining observed in Masson’s trichrome staining, indicating severely reduced lamellar number per bone area. Although OI type 4 also showed minimal red staining in Masson’s trichrome sections (Figure 2C and E), its birefringence pattern differed from that of OI type 1. Angular slice count analysis further distinguished these cases: OI types 1, 3, and 14 displayed relatively evenly distributed angular orientations, whereas OI type 4 showed three highly concentrated dominant orientation angles (Figure 4J).

### Bone mineralization and organic matrix composition profiles in OI cases evaluated by Raman spectroscopy

**Figure 5:**
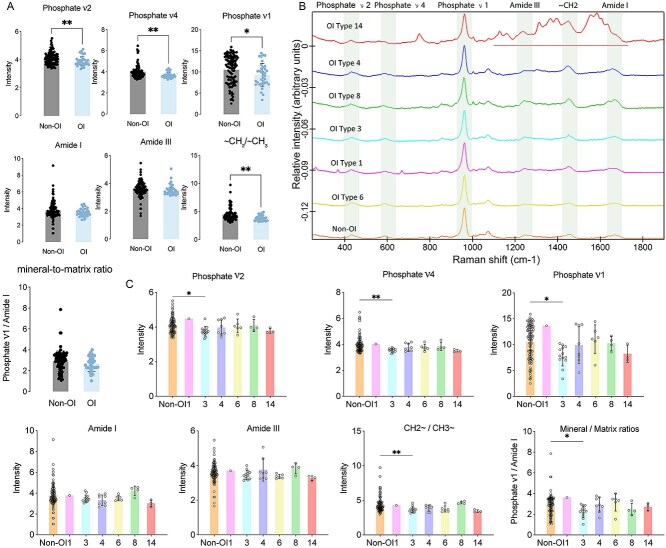
Raman spectroscopy analysis of bone mineral and organic matrix composition in non-OI and OI samples. (A) Bar plots showing the intensity of key Raman bands corresponding to bone mineral (phosphate ν1, ν2, and ν4), organic matrix components (amide I/III, CH₂/CH₃ groups), and mineral-to-matrix ratio. (B) Representative Raman spectra of bone samples from non-OI and OI individuals. Spectra were offset to minimize the impact of noise, and normalization was performed with reference to the amide III (1250 cm^−1^) band. The area marked with an underscoring line of OI type 14 indicates areas that are significantly different from non-OI samples. The shaded areas represent the different Raman spectral shift ranges corresponding to different components. (C) Bar plots showing the intensity of key Raman bands corresponding to bone mineral (phosphate ν1, ν2, and ν4), organic matrix components (amide I, amide III, and CH₂/CH₃ groups), and mineral-to-matrix ratio across non-OI controls and different OI cases. The pooled analysis should be considered as an overview, while the case-resolved analysis provides more detailed insight into heterogeneity within the OI cohort. Statistical significance is indicated where applicable (^**^p < .01, ^*^p < .05).

Raman spectral analysis was performed on the cortical bone of non-OI and OI patients to highlight key molecular vibrations associated with bone composition. Phosphate-related bands, ν2 (~431 cm^−1^), ν4 (~590 cm^−1^), and ν1 (~960 cm^−1^), were significantly reduced in OI samples compared to non-OI controls (Figure 5A, with *p*-values of .0062, .0017, and .0479, respectively). This reduction suggested decreased phosphate content, and a decrease in the content of hydroxyapatite crystals of OI bone, attributing to the different molecular vibrations, or movement of the phosphate groups. However, when comparing phosphate-related bands across OI cases, only OI type 3 showed a significant reduction compared to the non-OI controls (Figure 5C, with *p*-values of .0086, .0023, and .0217, respectively). The mineral-to-matrix ratio (phosphate ν1/Amide I), showed the same trend towards reduction in OI, but was not significantly reduced in general (Figure 5A) or in any of the OI types, apart from OI type 3 (Figure 5C). Amide I (~1666 cm^−1^) and Amide III (~1250 cm^−1^) bands, indicative of collagen secondary structure, exhibited no significant differences (Figure 5A). As Amide bands correspond to C=O (Amide I) and C-N and N-H (Amide III) peptide bonds of the collagen molecules, this indicates that the overall presence and secondary structure of collagen remains similar, though potential modifications in collagen cross-linking and organization may not be directly reflected in these Raman bands. Importantly, the ~CH₂/CH₃ band (~1450 cm^−1^) is associated with organic matrix composition and lipid content, and was significantly reduced (*p* < .01) in OI samples, which indicates the alkyl chains vibrational modes may be more heterogeneous, potentially leading to the collagen fibers being sparse and disordered. However, when comparing the ~CH₂/CH₃ band across OI cases, only OI type 3 showed a significant reduction compared to the non-OI controls (Figure 5C, with *p*-value .0067).

The representative Raman spectra of all bone samples suggests that OI type 14 was most distorted (Figure 5B), but this was not observed when comparing the specific mineral and organic matrix bands for OI type 14 to any of the other groups (Figure 5C). Additionally, case-specific secondary spectral features could be observed on the Raman spectra: for example, a cysteine ​​(~668 cm^−1^) signal peak in OI 1 bone samples, and a hydroxyproline (~872 cm^−1^) signal peak in OI 3 bone samples (Figure 5B). There was also a peak in the signal intensity at 756 cm^−1^ in the OI 14 samples. This has been attributed to B-type carbonate,^39^ but may also be related to the unknown component that also produces the bands present in the region between 1100 and 1700 cm^−1^ for this sample. Unfortunately, this region also obstructs the visibility of the 1070 cm^−1^ carbonate B band for verification of the band’s origin at 756 cm^−1^.

## Discussion

OI is a genetically and clinically heterogeneous disorder affecting bone integrity, yet prior studies have largely focused on the reduced bone phenotype rather than integrative histological–molecular characterization across multiple subtypes.^1^^,^^15^ While differences in bone mass, architecture, and collagen structure have been documented in OI patients with common COL1A1/COL1A2 mutations, especially in adult cohorts using DXA and high-resolution imaging,^40^ comparisons across multiple OI cases remain limited. Analyses that combine conventional histology with polarized light collagen assessment and Raman molecular profiling across diverse OI cases have not been extensively reported.

We observed that collagen organization was case-specifically altered in OI cortical bone, as evidenced by reduced lamellar thickness in OI types 1, 6, and 8, and increased green birefringence under polarized light microscopy in OI types 1 and 14. While non-OI bone displayed thick, well-organized lamellae with strong red-yellow birefringence (mean thickness: 7.5 μm with non-OI 1 and 4.4 μm with non-OI 2), OI samples showed precisely definable subtype-specific abnormalities. Specifically, lamellar thickness was severely reduced in OI types 1 and 6 (to 2.45 μm and 2.49 μm, respectively), whereas OI types 3 and 4 exhibited an intermediate degree of thinning (3.68 and 3.36 μm). These findings are in line with previous literature, as Chow et al. also demonstrated that human OI bone exhibits significantly thinner and more irregular lamellae compared with controls.^41^ Additionally, disturbed collagen organization observed in the present study agrees with earlier work^42^ showing that defects in collagen structure lead to disorganized extracellular matrix architecture in OI. Our observation of reduced lamellar thickness and altered birefringence across multiple OI cases is consistent with these reports and further extends them by demonstrating case-specific differences within OI cortical bone. In addition to case-specific collagen organization, our findings demonstrate pronounced differences in bone microarchitecture between non-OI and OI-affected cortical bone. Osteocyte density was increased in OI types 1, 6, 8, and 14. While Masson’s trichrome staining revealed OI samples exhibited varying degrees of immature less densely packed woven bone, compared to the relatively more continuous and organized lamellar regions in non-OI bone. The severity of woven bone organization increased progressively from OI type 1 to types 3, 6, 8, and 14. These observations align with previous reports showing that lamellae in moderate to severe OI are thinner and less structurally regular than in control bone,^41^ and that increasing disease severity is associated with persistence of immature woven bone structures.^15^

Raman spectroscopy revealed pronounced alterations in both mineral and organic matrix components of OI cortical bone compared with non-OI controls. Phosphate-related bands (ν1, ν2, and ν4) were significantly reduced, while Amide I and Amide III bands showed no significant differences, the CH₂/CH₃ band (~1450 cm^−1^) was markedly reduced, suggesting changes in the organic matrix composition. Moreover, Raman spectra demonstrated case-specific variations, including the most pronounced loss in OI type 3. OI type 3 also showed a decrease in mineral-to-matrix ratio; however, no significant differences were observed in overall of mineral-to-matrix ratio. This distinction is particularly relevant in OI, where previous human^43^ studies have consistently shown increased tissue mineralization and/or elevated mineral-to-matrix ratios despite impaired mechanical performance. For example, Imbert et al.^44^ demonstrated higher tissue mineral density, increased mineral-to-matrix ratio, and reduced crystallinity in human OI cortical bone, suggesting that OI bone contains relatively more mineral within a defective collagen matrix. Similarly, FTIR study^45^ in OI animal models has shown increased mineral-to-matrix ratios and altered crystal properties, supporting the concept that abnormal collagen promotes dysregulated mineral deposition rather than normal mineral maturation. A recent multiscale mouse study using Raman spectroscopy also reported that OI bone exhibits reduced collagen content and altered collagen distribution alongside a higher degree of mineralization compared with stiff and soft regions (increased ν1PO4^3−^/Amide I and ν1PO4^3−^/Amide III ratios in OI bone) of WT (wild-type bone).^46^ In contrast to these findings, the absence of significant differences in mineral-to-matrix ratios in the present study may reflect the localized nature of Raman spectroscopic measurements. Raman analysis probes a very small tissue volume at the bone surface and is therefore sensitive to local heterogeneity in matrix organization and mineralization. In OI, where bone tissue is highly heterogeneous and often contains regions of woven or newly formed bone, such localized measurements may capture site-specific variations associated with different stages of tissue maturation rather than global compositional differences. This may partly explain the discrepancy with previous studies that assessed bulk tissue properties or used techniques integrating larger tissue volumes. Woven bone is characterized by a relatively lower mineral content compared to lamellar bone, as newly formed osteoid initially consists primarily of collagen and water and undergoes progressive mineralization over time. Consistent with this, previous studies have demonstrated that mineral content in woven tissue is substantially lower than in mature lamellar bone, which contributes to its reduced stiffness.^47^ This is consistent with our study, in which a substantial proportion of woven bone was observed in the cortical bone samples from the OI group. Interestingly, despite our observed reductions in absolute phosphate-related signals, no significant differences were detected in mineral-to-matrix ratios between OI and control samples. Increased mineralization density in OI refers to spatial accumulation of mineral within an abnormal collagen matrix, whereas reduced phosphate-related Raman bands reflect altered mineral composition and crystallinity; these observations describe different aspects of bone mineralization and are therefore not contradictory. However, different Raman vibrational modes exhibit different sensitivities to the overall bone structure, and further comparative analysis is required to better interpret these differences. Additionally, case-specific abnormalities, such as the increased cysteine signal (~668 cm^−1^) in OI type 1 and the elevated hydroxyproline signal (~872 cm^−1^) in OI type 3. Another study using the same technique also found significant amounts of depositions of glycogen-rich, organic globules in OI patients,^48^ suggesting possible organic bone matrix composition alteration of OI patients.

This study has several limitations. The sample size was limited, and the cohort was heterogeneous in terms of age, anatomical sampling site, and clinical background; therefore, comparisons between OI cases should be considered exploratory. Consequently, this study is presented as a series of cases and is not statistically powered to draw definitive conclusions regarding differences between individual OI cases. In addition, the non-OI samples represent a clinical reference group rather than ideal healthy controls, as they were obtained from corrective surgeries. Due to ethical constraints, obtaining strictly healthy pediatric cortical bone is impossible; thus, our non-OI samples were opportunistically sourced from surgical waste of individuals requiring corrective osteotomies for other skeletal conditions (eg, achondroplasia). These factors may influence bone microstructure and partly explain the presence of less organized matrix in control samples. Additionally, the patient with OI type 14 carried mosaic Turner syndrome (45,X/46,XX) in addition to the homozygous pathogenic TMEM38B variant. As Turner syndrome has been associated with altered bone metabolism, reduced bone mass, and skeletal abnormalities independent of OI,^49^ its presence may have contributed to the observed bone tissue phenotype and should therefore be considered a potential confounding factor when interpreting findings from this case. Furthermore, because the surgical bone specimens were obtained during corrective osteotomies, they may originate near historical fracture sites. Consequently, the high woven bone percentage (43%-91%) observed across all samples, including non-OI controls, likely reflects localized fracture repair and mechanical adaptation rather than solely the baseline genetic phenotype, representing a potential mixture of both processes. We observed a disproportionately high amount of woven bone (~80%) in mild OI type 1 samples, whereas severe OI type 3 samples exhibited substantial dense lamellar bone (57%). According to Shapiro et al.,^15^ the structural maturation of OI bone generally inversely correlates with disease severity; milder forms (eg, type I) are expected to form well-developed lamellar bone approaching normal structural compaction, while more severe forms are characterized by a greater persistence of immature woven bone. Despite these limitations, this study provides valuable multiscale characterization of cortical bone across genetically diverse OI cases using an integrated histological and molecular approach. Access to pediatric bone tissue from rare OI genotypes is exceptionally limited, and few prior studies^15^^,^^44^^,^^48^ have combined histology, polarized light microscopy, and Raman spectroscopy within the same cohort. Therefore, although the findings should be interpreted cautiously, the present work offers important exploratory insight into case-specific alterations in bone matrix organization and composition, and establishes a framework for future larger-scale mechanistic studies in OI.

In conclusion, this study highlights the critical role of collagen organization and bone mineralization in maintaining bone integrity and provides a detailed characterization of the histological and molecular alterations in different OI patients. The case specific disorganization of collagen fibers and dysfunctional mineralization observed in OI-affected bone underscore the complexity of this condition. By integrating histological and molecular analyses, this study contributes to the understanding of the structural and molecular underpinnings of bone fragility across diverse OI cases.

## Supplementary Material

Supplementary_Figure_1_ziag111

Supplementary_figure_2_ziag111

## Data Availability

The data supporting the findings of this study are available from the corresponding author upon reasonable request.
