# Supplementary material for: Histological and molecular characterization of bone integrity in osteogenesis imperfecta: a case series across genetic subtypes
Source: JBMR Plus. 2026 Jul 12;10(8):ziag111. doi: 10.1093/jbmrpl/ziag111 (PMC13411277; doi:10.1093/jbmrpl/ziag111)
Supplement: Supplementary_Figure_1_ziag111 [file supplementary_figure_1_ziag111.pdf]

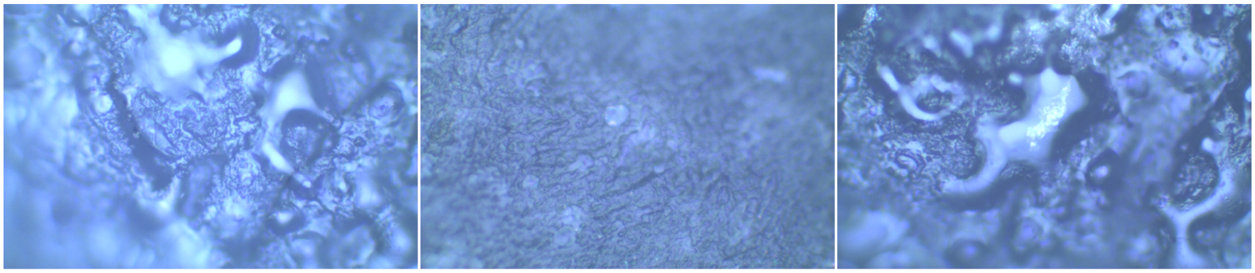

Supplementary Figure 1. Representative optical image of bone specimens during Raman spectroscopy acquisition.

Representative image showing cortical bone fragments placed directly on a glass slide for Raman spectroscopic measurements. Samples were analyzed in their native, non-embedded and non-decalcified state to preserve the intrinsic mineral and organic matrix composition. Raman measurements were performed on selected regions of the bone surface, avoiding visible artifacts such as cracks and voids, and covering both relatively homogeneous and heterogeneous areas of the matrix. The image illustrates the general morphology of the analyzed fragments and the experimental setup used for spectral acquisition.
