# Supplementary figures and images for: Histological and molecular characterization of bone integrity in osteogenesis imperfecta: a case series across genetic subtypes
Source: JBMR Plus. 2026 Jul 12;10(8):ziag111. doi: 10.1093/jbmrpl/ziag111 (PMC13411277; doi:10.1093/jbmrpl/ziag111)

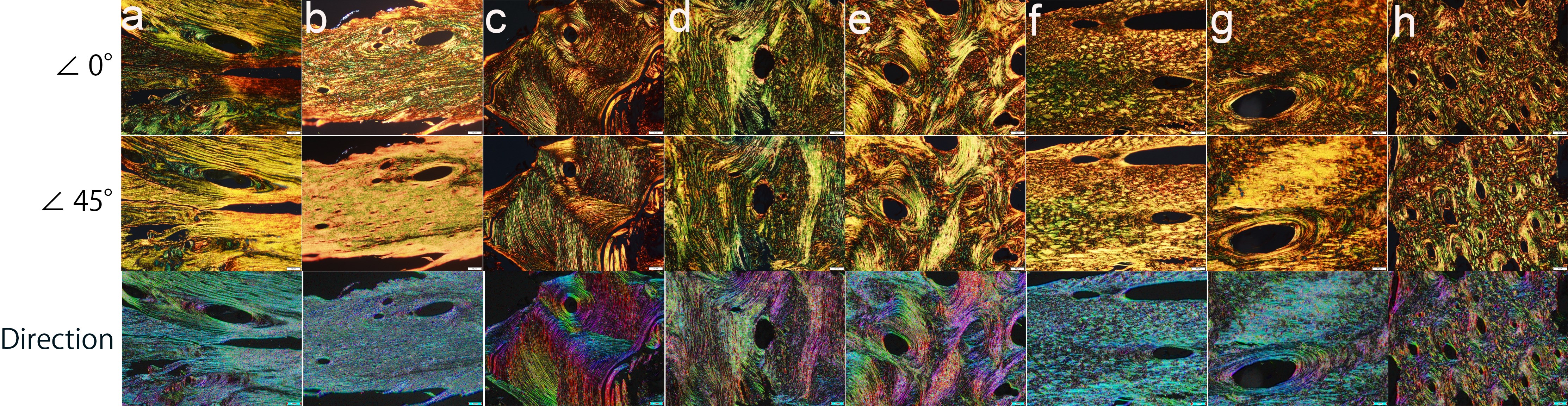

Supplement: Supplementary_figure_2_ziag111 [file supplementary_figure_2_ziag111.jpeg]
